# 妊娠期合并伴NPM1突变急性髓系白血病自发缓解1例

**DOI:** 10.3760/cma.j.cn121090-20230905-00103

**Published:** 2024-09

**Authors:** 冉 张, 桐硕 张, 玉玲 潘, 莉莉 王, 瑷博 逄, 立萍 窦, 彧 靖

**Affiliations:** 1 解放军总医院第五医学中心血液病医学部，北京 100071 Senior Department of Hematology, the Fifth Medical Center, Chinese PLA General Hospital, Beijing 100071, China; 2 解放军总医院第一医学中心血液科，北京 100853 Department of Hematology, the First Medical Center, Chinese PLA General Hospital, Beijing 100853, China; 3 武警江苏总队医院检验与病理科，扬州 225003 Department of Laboratory Medicine and Pathology, Jiangsu Provincial Corps Hospital of Chinese People's Armed Police Force, Yangzhou 225003, China; 4 解放军总医院第一医学中心检验科，北京 100853 Clinical Laboratory, the First Medical Center, Chinese PLA General Hospital, Beijing 100853, China; 5 解放军医学院，北京 100853 Medical School of Chinese PLA, Beijing 100853, China

患者，女，30岁。2015年9月（妊娠30周左右）周身出现红色皮疹，无瘙痒及疼痛不适，2015年10月初皮肤现散在出血点，2015年10月15日于当地医院产检（妊娠34～35周）发现血常规异常：WBC 5.13×10^9^/L，HGB 83 g/L，PLT 43×10^9^/L。给予输注人免疫球蛋白20 g，血小板2 U治疗后，血小板一过性恢复至正常。后监测血常规，外周血三系细胞逐渐下降。2015年10月19日晨起左侧面部麻木，味觉消失，嘴角左偏，予甲钴胺、维生素B_1_、维生素B_12_营养神经治疗，并予每日口服醋酸地塞米松片15 mg治疗共7 d，间断输血及血小板，输血前加用地塞米松磷酸钠注射液预防过敏反应，血常规指标改善不明显。2015年10月23日行骨髓穿刺检查，骨髓象：增生活跃，粒红两系增生受抑，单核系统增生，约占85.5％，其中幼稚单核细胞占76％；全片未见巨核细胞，血小板少见。外周血涂片示白细胞正常，粒细胞减少，核左移，单核细胞多，约占61％，其中幼稚单核细胞占53％；血小板少；POX染色阴性或者弱阳性；α-NBE染色阳性，可被NAF抑制。诊断考虑急性白血病，不排除急性髓系白血病（AML）-M_5b_。未行骨髓活检及流式细胞术检测，同时未做基因检测、染色体检查。2015年10月26日行剖宫产手术，硬膜外麻醉取出少量脑脊液检查，发现镜下易见单核细胞，可见少量幼稚单核细胞及红细胞。2015年11月3日，因紫癜1个月、面瘫半个月入我院。否认家族遗传史。查体：面瘫面容，口角左偏，颈部右侧可触及直径约1 cm大小淋巴结，质硬，活动度差，无压痛及粘连。皮肤黏膜未见出血点、瘀斑、蜘蛛痣。双肺呼吸音清，未闻及干、湿啰音。心率82次/min，律齐，各瓣膜区未闻及杂音。臀部可见妊娠纹，下腹部可见长约10 cm横行切口，愈合好。四肢活动正常。双侧病理征阴性。2015年11月3日行PET-CT结果提示：①子宫弥漫不均高代谢（SUVmax5.2），下腹壁片状高代谢（SUVmax3.9），以上均首先考虑术后改变。②食管条形高代谢，多考虑生理或炎性可能。③左室心肌结节样高代谢灶，考虑生理性摄取。④右肺水平裂旁小结节，无异常。⑤副脾结节，肝右叶囊肿。⑥脑部未见明显高代谢病变。2015年11月4日行骨髓穿刺细胞学检查，骨髓象：①增生明显活跃，髓系原始幼稚单核细胞占9.2％（图 1 A1、A2）。②复习原始涂片（2015年10月23日当地医院借至我院会诊），髓系原始幼稚单核细胞占80％，符合AML骨髓象，多考虑AML-M_5_。免疫分型：检出4.22％的幼稚单核细胞，强阳性表达CD33、CD64、CD15、HLA-DR，弱阳性表达CD13、CD11b，不表达CD34、CD117、CD16、CD14。NPM1基因定量：201％（表1）。染色体核型：46,XX[20]。行腰椎穿刺术2次，每次鞘内注射地塞米松磷酸钠注射液共5 mg，脑脊液常规、生化未见明显异常，脑脊液未找见白血病细胞。诊断：①AML-M_5_伴NPM1突变，低危；②剖腹产术后；③周围性面瘫。

**图1:**
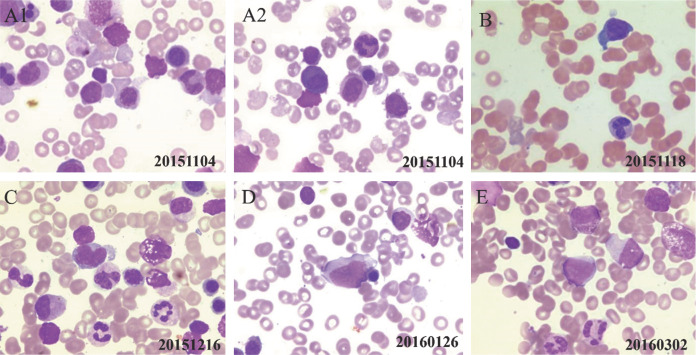
该例妊娠期合并伴NPM1突变急性髓系白血病患者初诊（A1、A2）及自发缓解前后（B～E）骨髓细胞形态（1000×） **注** 图片右下角标为行骨髓穿刺时间

**表1:** 该例妊娠期合并伴NPM1突变急性髓系白血病患者历次骨髓穿刺检查结果

检测日期	原始细胞比例 （%）	免疫分型异常细胞比例 （%）	NPM1基因定量^a^ （%）	染色体
2015-10-23（当地医院）	76.0	–	–	–
2015-11-04（本院）	9.2	4.22	201.00	46,XX[20]
2015-11-18	2.4	0.56	–	–
2015-12-16	3.6	0.03	0.85	–
2016-01-26	2.0	0.01	0.28	–
2016-03-02	2.8	0.02	0.31	–
2017-01-16	阴性	阴性	阴性	–

**注** –：未做。^a^我中心血液科实验室采用实时荧光定量PCR法检测NPM1基因A、B、D三种主要突变体，以突变型拷贝数与内参基因拷贝数的比值作为NPM1突变的转录水平，称“NPM1 A型突变转录本水平”，简称“NPM1基因定量”

入院后进行神经内科会诊，予营养神经治疗，每日静脉滴注单唾液酸四己糖神经节苷脂40 mg×20 d，静脉滴注鼠神经生长因子30 mg×7 d，肌肉注射腺苷钴胺1.5 mg×5 d，并予面部局部理疗5 d纠正面瘫。予静脉滴注莫西沙星0.4 g×5 d，静脉滴注重组人血小板生成素1.5万U×7 d。予输注去白细胞红细胞4 U（600 ml），去白细胞血小板2 U（500 ml）对症支持治疗，其中输血前应用地塞米松共2次，总量4 mg。未行化疗、放疗及免疫治疗。

2015年11月18日复查，骨髓象：增生极度活跃，髓系原始幼稚单核细胞平均占2.4％（图1B）；免疫分型：检出0.56％的幼稚髓系细胞，异常表达CD34、HLA-DR、CD117、CD7。2015年12月16日至2016年3月2日多次复查，骨髓象、免疫分型及NPM1基因定量结果见表1，历次骨髓象见图1C～E。2017年1月16日于我院海南分院就诊，骨髓象：增生活跃，铁染色示缺铁；流式细胞术可见CD34^+^细胞占有核细胞总数0.5％，其免疫表型未见明显异常；粒细胞相对比例正常，免疫表型CD13、CD16、CD15、CD11b未见明显表达紊乱，送检标本中未检测到明显急性白血病和高危骨髓增生异常综合征相关免疫表型异常证据。未检测到NPM1基因突变。末次随访（2023年9月），患者状态好，无白血病复发临床表现。血常规正常，2017年以后未再行骨髓穿刺检查。

讨论：妊娠合并NPM1基因突变AML发生自发缓解的病例极其罕见，本例患者诊断AML，终止妊娠后，未行放化疗，确诊后15个月后白血病微小残留转阴达持续完全缓解状态。病程中未出现发热、严重感染，输注大量血制品及应用大量糖皮质激素，病情逐渐好转是从解除妊娠开始，考虑白血病自发缓解与妊娠终止相关，推断此类白血病患者的白血病细胞为雌激素依赖性的可能性较大，不能排除综合因素。不同的基因突变导致的异常克隆生存能力不一，自发缓解是否与白血病类型及特殊基因突变相关，须大样本进一步证实。

